# Upregulation of HDAC9 in hippocampal neurons mediates depression-like behaviours by inhibiting ANXA2 degradation

**DOI:** 10.1007/s00018-023-04945-y

**Published:** 2023-09-10

**Authors:** Yunjian Dai, Taofeng Wei, Yuwen Huang, Yun Bei, Haoran Lin, Zexu Shen, Lingyan Yu, Mingdong Yang, Huimin Xu, Wei He, Zheng Lin, Haibin Dai

**Affiliations:** 1 Department of Pharmacy, Second Affiliated Hospital, Zhejiang University School of Medicine, 88 Jiefang Road, Hangzhou, 310009 China; 2 Department of Psychiatry, Second Affiliated Hospital, Zhejiang University School of Medicine, Hangzhou, 310009 China

**Keywords:** Histone deacetylase 9, Depression, Annexin A2, Hippocampal neurons, Acetylation, Ubiquitination

## Abstract

Major depressive disorder (MDD) is a pervasive and devastating mental disease. Broad spectrum histone deacetylase (HDAC) inhibitors are considered to have potential for the treatment of depressive phenotype in mice. However, due to its non-specific inhibition, it has extensive side effects and can not be used in clinical treatment of MDD. Therefore, finding specific HDAC subtypes that play a major role in the etiology of MDD is the key to develop corresponding specific inhibitors as antidepressants in the future. Copy number variation in HDAC9 gene is thought to be associated with the etiology of some psychiatric disorders. Herein, we found that HDAC9 was highly expressed in the hippocampus of chronic restraint stress (CRS) mouse model of depression. Upregulation of HDAC9 expression in hippocampal neurons of mice induced depression-like phenotypes, including anhedonia, helplessness, decreased dendritic spine density, and neuronal hypoexcitability. Moreover, knockdown or knockout of HDAC9 in hippocampal neurons alleviated depression-like phenotypes caused by chronic restraint stress (CRS) in WT mice. Importantly, using immunoprecipitation–mass spectrometry (IP–MS), we further found that Annexin A2 (ANXA2) was coupled to and deacetylated by HDAC9. This coupling resulted in the inhibition of ubiquitinated ANXA2 degradation and then mediates depression-like behavior. Overall, we discovered a previously unrecognized role for HDAC9 in hippocampal neurons in the pathogenesis of depression, indicating that inhibition of HDAC9 might be a promising clinical strategy for the treatment of depressive disorders.

**Supplementary Information:**

The online version contains supplementary material available at 10.1007/s00018-023-04945-y.

## Introduction

Depression is a common but serious mood disorder with a major impact on quality of life. For example, the rate of suicide among patients with depression ranges between 4.0 % and 10.6% [1]. The treatment of depression, especially major depressive disorder (MDD), remains inadequate. Most antidepressants currently in the clinic require long-term treatment, and risk of relapse after treatment is high. Developing the next generation of drugs requires deeper understanding of the molecules and pathways that contribute to the disorder.

Understanding the pathogenesis of depression requires clarifying the potential contribution of histone deacetylases (HDACs). Traditionally researched for their role in deacetylating histones and thereby silencing gene transcription [2, 3], some of these enzymes, mainly class II HDACs, deacetylate non-histone proteins, thereby regulating the activity of proteins whose localization or function may depend on posttranslational acetylation [4–8]. Altered HDAC expression has long been linked to carcinogenesis [9], and more recent studies have linked it to depressive episodes [10, 11]. Sodium butyrate, which inhibits various HDACs, exerts therapeutic effects in a mouse model of depression [12]. Given the widespread role of HDACs in biological processes [13], developing HDAC inhibitors as antidepressants will require inhibiting specifically the HDACs that contribute to MDD in order to minimize side effects. However, little is known about the expression patterns and roles of different HDACs in depression.

To begin to address these questions, we focused on the HDAC9 because it may participate in synaptic plasticity and memory formation [14], its gene shows copy number variations in patients with schizophrenia [15]. In the present study, we monitored how HDAC9 overexpression or knockdown in hippocampal neurons affected depression-like behaviours in mice, finding evidence that HDAC9 upregulation may contribute to MDD. We also explored potential binding partners of HDAC9 and found evidence that HDAC9 regulates the acetylation and ubiquitination of ANXA2. HDAC9 upregulation may protect ANXA2 from ubiquitin-dependent degradation, thereby contributing to depression-like symptoms. Our results provide the first evidence implicating HDAC9 in hippocampal neurons may play a role in pathophysiology of depression, implying its usefulness as a therapeutic target.

## Materials and methods

### Animals

C57BL/6 mice and HDAC9 conditional knockout (CKO) mice (Laboratory Animal Center of Zhejiang University, Hangzhou, China) were used in this study. The mice were kept in clean cages on a 12-h light–dark cycle. The experimental mice (2–3 months old) were used for behavioral tests.

### Animal model of depression

Male C57BL/6 mice (8 weeks old) were obtained from the Laboratory Animal Centre of Zhejiang University (Hangzhou, China) and kept in clean cages on a 12-h light–dark cycle. Mice were subjected to chronic restraint stress (CRS) as described [16] in order to induce a depression-like phenotype. First, animals were allowed to acclimate in the behavioural testing room for one week on a 12-h light–dark cycle with free access to food and water. At the end of this period, animal weight, sucrose preference, water preference, and performance in the open field test (OFT) (see below) were measured, then animals with similar assessments were assigned to control and CRS groups. For CRS group, mice were transferred to the experimental room and individually placed in a 50-mL centrifuge tube with multiple holes and restrained for 6 h beginning around 13:00 h per day for 21 days. For Control group, mice were transferred to the experimental room without being exposed to restraint stress, then returned to the feeding room after 6 h. All tubes were cleaned with water and sprayed with a 75% alcohol solution each day. Depression-like behaviours were evaluated at 24 h after the final stress exposure, then brain samples were taken 24 h after the final behavioral test (see below).

### Generation of HDAC9 conditional knockout (CKO) mice

HDAC9^flox/flox^ mice were obtained from Cyagen Biotechnology (Guangzhou, China). HDAC9^flox/flox^ mice were crossed with CaMKIIα-Cre mice to obtain HDAC9^flox/flox^/CaMKIIα Cre mice, hereafter referred to as “HDAC9 CKO mice”. These animals had HDAC9 knockout in forebrain neurons. CKO mice were maintained under similar conditions as C57BL/6 mice.

### Generation of recombinant adeno-associated viruses to overexpress or knockdown HDAC9 in hippocampal neurons

Genechem company (Shanghai,China) created adeno-associated virus encoding mouse HDAC9 (AAV2/9-CAG-Hdac9-3xFlag-SV40, hereafter “AAV-HDAC9”). A corresponding “empty” adeno-associated virus was created as a negative control (hereafter “AAV-CON”). The two viruses were injected at 2.31 × 10^13^ or 3.17 × 10^12^ vector genomes per mL bilaterally into the dorsal hippocampus of male CaMKIIα-Cre mice (2 months old).

Genechem company (Shanghai, China) created adeno-associated virus encoding short hairpin RNA (shRNA) targeting HDAC9 in hippocampal neurons (AAV2/9-CaMKIIα-eGFP-3xFLAG-miR30shRNA, hereafter “AAV-shHDAC9”). The corresponding “empty” virus was created as a negative control (AAV2/9-CaMKIIα-eGFP, hereafter “AAV-shCON”). The viruses were injected at 1.0 × 10^12^ vector genomes per mL bilaterally into the dorsal hippocampus of male CRS mice (2 months old).

Genechem company (Shanghai, China) created adeno-associated virus encoding small guide RNA (sgRNA) targeting ANXA2 in hippocampus (AAV2/9-saCAS9-T2A-eGFP-ANXA2-sgRNA, hereafter “AAV-sgANXA2”). The corresponding “empty” virus was created as a negative control (AAV2/9-eGFP, hereafter “AAV-sgCON”). The viruses were injected at 3.44 × 10^12^ vector genomes per mL bilaterally into the dorsal hippocampus of male CRS mice (2 months old).

### Stereotaxic injection of recombinant viruses

Mice were first anesthetized by intraperitoneal injection of 1% sodium pentobarbital (60 mg/kg) and then fixed on a stereotaxic apparatus (Stoelting, USA). Virus (0.7 μL) was injected into the corresponding sites of the CA1 region of hippocampus (AP: − 2 mm, ML: ± 1.3 mm, DV: − 1.6 mm) through a 1μL microsyringe (Shanghai Gaoge Industry and Trade, Shanghai, China). The injection speed was maintained at 0.07 μL/min using a syringe pump. After virus was injected, the needle was held in position for 10 min, then slowly withdrawn [17].

At 4 weeks later, the accuracy of the injection site was confirmed by examining immunofluorescence. Only mice showing expression of eGFP or Flag in hippocampal neurons were subjected to further analyses. Electrophysiological recording was performed 3–4 weeks after virus injection, while behavioural tests were performed 4–6 weeks after injection.

### Behavioural tests

#### OFT

Mice were placed in the centre of a test chamber measuring 40 × 40 cm, and the paths of the mice were recorded with a video camera to evaluate the total distance travelled. The chamber was cleaned with a 75% alcohol solution after each test.

#### Forced swimming test (FST)

Mice were placed in a cylindrical glass tank of height 20 cm and diameter 14 cm. The depth of the water in the tank was 6, 10, or 15 cm according to the experimental requirements, and the water temperature was approximately 25 °C. The mice were allowed to swim in the tank for 6 min, and the cumulative immobility time during the last five minutes was recorded.

#### Tail suspension test (TST)

Mice were suspended by their tails with their heads approximately 35 cm above the ground. The cumulative immobility time during 5 min was recorded as a measure of helplessness.

#### Sucrose preference test (SPT)

First, mice were adapted to drinking water that contained sugar. Each cage was equipped with two water bottles, one containing pure water and one containing 1% sucrose in water. The mice were allowed to adapt for three days. The positions of the bottles were changed daily to prevent positional preference. For the SPT, each mouse was fasted for 24 h and then given a preweighed bottle of pure water and a preweighed bottle of 1% sucrose in water. After 1 h, the weights of the bottles were measured. The amount of pure water or sucrose water consumed by the mice was used to calculate the sucrose preference of each mouse:sucrose preference (%) = sucrose water intake/(sucrose water intake + pure water intake). This preference served as a measure of anhedonia.

### Golgi staining and measurement of dendritic spine density

We used Golgi staining to measure the density of dendritic spines on hippocampal neurons in mice following their behavioural testing (n = 3 per group). Mice brains were collected and processed for the Golgi staining according to the protocol provided from the FD Rapid Golgi Stain Kit (PK401, FD NeuroTechnologies). The number of dendritic spines was analysed using Image-Pro Plus 6.0 as described [18], with modifications. Spines were examined on dendrites of CA1 neurons that satisfied the following criteria: (1) presence of untruncated dendrites, (2) dark and consistent Golgi staining throughout all dendrites, and (3) visual separability from neighbouring neurons. We counted the number of dendritic spines along the second or third dendritic branch at distances from 30 to 90 µm from the cell body, in images obtained at 400× magnification. The “density of dendritic spines” was defined as the number of spines per 10 µm and was calculated as density = number of dendritic spines/dendritic length × 10. For each group, at least three dendritic segments per neuron in at least five pyramidal neurons were analysed from each mouse.

Spines were classified as thin, if they had a long, slender neck and small, rounded head < 2 μm long; mushroom, if they had a well-defined, thick neck and a voluminous head > 0.6 μm wide; or stubby, if they were short and thick, without a distinguishable neck, and a ratio of length: width = 1.

### Electrophysiology of brain slices

In vitro electrophysiology was performed according to our previous study [19]. After mice completed behavioural testing (see above), they were anesthetized by intraperitoneal injection of sodium pentobarbital and their brains were immediately removed and immersed at 0 °C in cutting solution. Coronal slices that were 300 µm thick and that included the hippocampus were prepared using a vibratome (VT1000S, Leica, Wetzlar, Germany) and incubated for 1 h at 25 °C in artificial cerebrospinal fluid (ACSF), which contained (in mM) NaCl (125), KCl (3.5), NaH_2_PO_4_ (1.25), MgCl_2_ (0.5), NaHCO_3_ (26), dextrose (25), and CaCl_2_ (1). Then the slices were transferred into a recording chamber at 25 °C for in vitro electrophysiological recording. Patch electrodes with tip resistances of 4–7 MΩ were filled with internal solution containing (in mM) CsCH_3_SO_3_ (100), KCl (20), HEPES (10), Mg-ATP (4), Tris-GTP (0.3), Tris-2-phosphocreatine (7), and QX-314 (3). Neurons in the CA1 of the hippocampus were visualized with a 40 × water-immersion lens and recorded with an EPC10 amplifier (HEKA Instruments, Lambrecht, Germany). Spontaneous excitatory postsynaptic currents (sEPSCs) were recorded at a holding potential of − 60 mV. Individual events were counted and analysed with MiniAnalysis 6.0.3.

### Western blot analysis of hippocampal proteins

After mice completed behavioural testing (see above), hippocampus was rapidly isolated, homogenized in lysis buffer supplemented with protease inhibitor cocktail, and centrifuged. The supernatant was collected, the protein concentration was measured using a bicinchoninic acid assay kit (Beyotime, Shanghai, China), and equal amounts of protein (30 μg) were electrophoretically separated on 8–15% gradient SDS-PAGE gels, then transferred to polyvinylidene difluoride membranes. Membranes were incubated with primary antibodies against the following: HDAC1 (1:5000, 66085-1-Ig, Proteintech, Wuhan, China), HDAC2 (1:5000, 12922-3-AP, Proteintech, Wuhan, China), HDAC4 (1:1000, 66838-1-Ig, Proteintech, Wuhan, China), HDAC5 (1:500, A01230-6, BOSTER, Wuhan, China), HDAC9 (1:1000, AP1109a, Abcepta, Guangzhou, China), β-tubulin (1:3000, 10094-1-AP, Proteintech, Wuhan, China), GAPDH (1:5000, 10494-1-AP, Proteintech), and ANXA2 (1:5000, 60051-1-lg, Proteintech). Next, membranes were incubated with horseradish peroxidase-conjugated anti-rabbit/mouse immunoglobulin G (IgG) as secondary antibody (1:5000, 70-GAR007/70-GAM007, MultiSciences, Hangzhou, China). Protein band densities were quantified using Image J.

### Quantitation of mRNA levels of hippocampal proteins

Total RNA was extracted from the hippocampi isolated as described above using the EZ-10 Total RNA Mini-Prep Kit (Sangon Biotech, Shanghai, China). An mRNA reverse transcription system was configured to ensure that the concentration and content of each group of mRNAs added to each system were consistent, then the mRNA was reverse-transcribed to obtain cDNA using the primers (Table S1). Levels of target mRNAs were quantified relative to levels of GAPDH.

### Immunoprecipitation of hippocampal tissues

Cut the tissue into small fragments, add Cell lysis buffer for Western and IP (P0013, Beyotime, Shanghai, China) and mix well. Homogenize with a glass homogenizer, centrifuge 12000*g* for 25 min, and take the supernatant. 400 μg tissue lysates were incubated with the appropriate antibodies at 4 °C overnight and subsequently with Protein A/G PLUS-Agarose (sc-2003, Santa, Dallas, USA) for 2 h at 4 °C. Centrifuge at 2500 rpm at 4 °C for 5 min to remove the supernatant. Thereafter, the protein-antibody complexes were washed three times at 4 °C with cold lysis buffer and eluted with 2× SDS sample buffer by boiling for 5 min. Take the supernatant after centrifugation and discard the sediment. The obtained supernatant can be used for subsequent WB analysis.

### Immunochemistry of brain sections

One day after behavioural testing, mice were anesthetized with sodium pentobarbital and perfused with 4% paraformaldehyde. The brains were isolated, fixed in paraformaldehyde overnight, dehydrated in sucrose solution, and then cut into serial coronal sections 30 μm thick using a cryostat. The sections were incubated with antibodies against the following proteins: HDAC9 (1:100, AP1109b, Abcepta), NeuN (1:200, ab104224, abcam, USA), GFAP (1:200, ab53554, abcam), Iba1 (1:200, ab5076, abcam), and CaMKIIα (1:100, ab22609, abcam). Then the sections were incubated with fluorescently labelled secondary antibody conjugated to Alexafluor 488 or 594 (1:500, Proteintech). Images were captured using a laser scanning confocal microscope.

### Statistical analysis

All data are presented as the mean ± standard error of the mean (SEM). The sample size for each experiment is provided in figure legends. All statistical analyses were performed using GraphPad Prism version 8. Differences between two groups were assessed for significance using an unpaired two-samples t test. Differences among three or more groups were assessed using one-way ANOVA with Tukey’s post hoc test (ANOVA).

## Results

### HDAC9 is upregulated in CRS mice

**Fig. 1:**
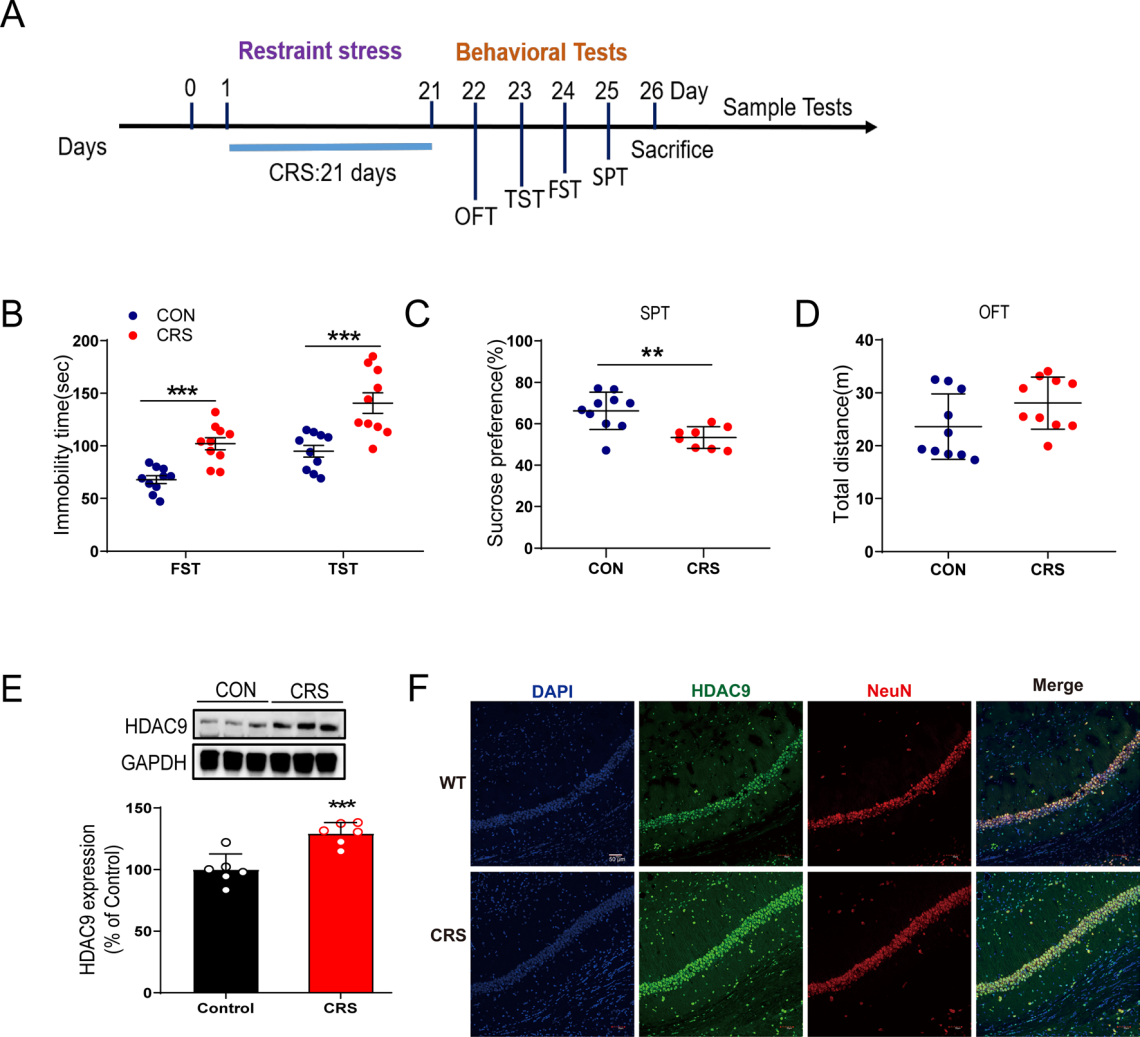
HDAC9 expression is increased in mice exposed to chronic restraint stress (CRS). **A** Timeline of CRS, behavioural tests, and immunohistochemistry. **B** Forced swimming test (FST) and tail suspension test (TST) [n = 10 CRS mice and 10 control (CON) mice; FST, p < 0.0001; TST, *p* = 0.0007]. **C** Sucrose preference test (SPT) (n = 10 CRS mice and 10 CON mice; *p* = 0.0026). **D** Open-field test (OFT) (n = 10 CRS mice and 10 CON mice; *p* = 0.0917). **E** Expression of hippocampal HDAC9 (n = 6 mice per group; *p* = 0.001). **F** Representative double immunostaining of NeuN and HDAC9, showing high expression of HDAC9 in neurons in the CA1 region in CRS mice. *p < 0.05, **p < 0.01, ***p < 0.001. Unpaired two-tailed Student’s t test. Data are shown as mean ± standard error of the mean

We tested the effect of CRS on the expression of HDACs in the hippocampus. Adult mice were exposed to CRS for 21 days (Fig. 1A), the contents of HDAC9 were increased in the hippocampus of CRS mice, whereas other HDACs were not affected (Fig. S1 and 1E). CRS mice showed longer immobility times in the FST and TST than control animals (Fig. 1B), as well as lower preference for sucrose solution in the SPT (Fig. 1C). In the OFT, the two groups showed similar locomotor ability (Fig. 1D).

In addition, the content of HDAC9 in the prefrontal cortex and amygdala remains unchanged (Fig. S2A). Within the CRS hippocampus, HDAC9 was overexpressed in neurons, identified based on their expression of the neuronal marker NeuN, but not in microglia or astrocytes, identified by their respective expression of Iba1 or GFAP (Fig. 1F, Fig. S2B–C). Moreover, we found that after experiencing CRS, there was no significant change in the distribution of HDAC9 in neurons of other depression related brain regions such as Lateral hypothalamus (LH), Lateral habenula (LHB) and Prelimbic cortex (PrL) (Fig. S3).

**Fig. 2:**
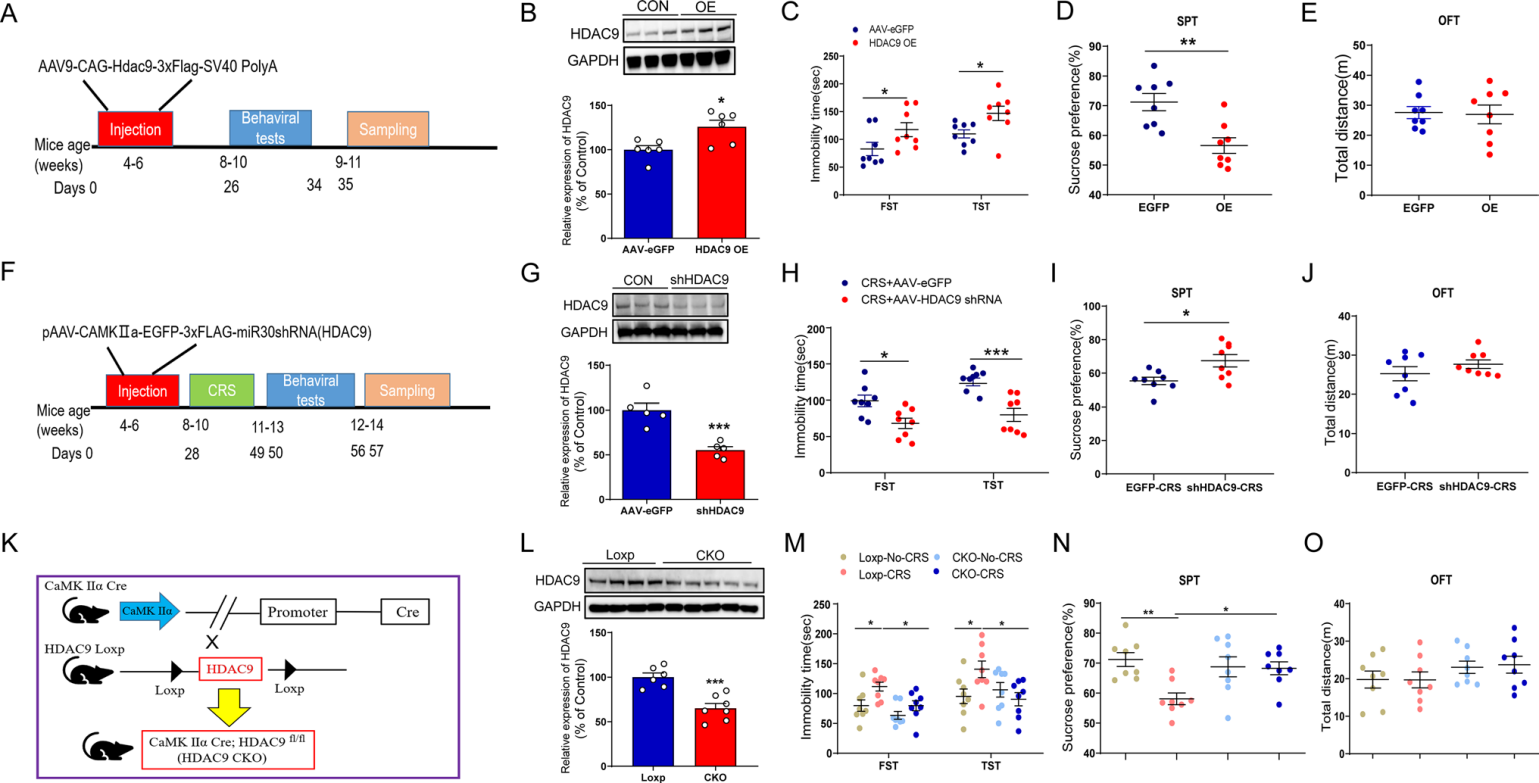
HDAC9 levels in the hippocampus affect depression-like behaviours in mice. **A**–**E** Experiments involving mice overexpressing HDAC9 in the hippocampus. **A** Timeline of injection of adeno-associated virus (AAV), behavioural tests, and western blotting. **B** Expression of hippocampal HDAC9 in mice injected with AAV-HDAC9 (n = 6 mice per group; *p* = 0.0137). **C** Forced swimming test (FST) and tail suspension test (TST) in mice injected with AAV-HDAC9 in the hippocampus. (n = 8 for AAV-HDAC9-injected mice, n = 8 for AAV-eGFP-injected mice; FST, *p* = 0.0414; TST, *p* = 0.0306). **D** Sucrose preference test (SPT) in mice injected with AAV-HDAC9 in the hippocampus (n = 8 for AAV-HDAC9-injected mice, n = 8 for AAV-eGFP-injected mice; *p* = 0.0026). **E** Open-field test (OFT) in mice injected with AAV-HDAC9 in the hippocampus (n = 8 for AAV-HDAC9-injected mice, n = 8 for AAV-eGFP-injected mice; *p* = 0.8668). **F** Timeline of AAV injection, behavioural tests, and western blot. **G** Expression of hippocampal HDAC9 protein in mice injected with AAV-shHDAC9 (n = 5 mice per group; *p* = 0.0009). **H** FST and TST in CRS mice injected with AAV-shHDAC9 (n = 8 mice injected with AAV-shHDAC9 and exposed to CRS; n = 8 mice injected with AAV-eGFP and exposed to CRS; FST, *p* = 0.0114; TST, *p* = 0.0007). **I** SPT in CRS mice injected with AAV-shHDAC9 (n = 8 mice injected with AAV-shHDAC9 and exposed to CRS, n = 8 mice injected with AAV-eGFP and exposed to CRS; *p* = 0.0143). **J** OFT in CRS mice injected with AAV-shHDAC9 (n = 8 mice injected with AAV-shHDAC9 and exposed to CRS, n = 8 mice injected with AAV-eGFP and exposed to CRS; *p* = 0.2727). **K** Diagram of the strategy used to generate HDAC9 conditional knockout (CKO) mice. **L** Expression of hippocampal HDAC9 in HDAC9 CKO mice (n = 6 for Loxp mice, n = 7 for HDAC9 CKO mice, *p* = 0.0005). **M** FST and TST in HDAC9 CKO mice subjected to CRS (n = 8 in each of the four groups; FST, *p* = 0.0433 for Loxp-CRS vs. Loxp-No-CRS, *p* = 0.0412 for Loxp-CRS vs. CKO-CRS; TST, *p* = 0.0476 for Loxp-CRS vs. Loxp-No-CRS, *p* = 0.0315 for Loxp-CRS vs. CKO-CRS). **N** SPT in HDAC9 CKO mice subjected to CRS (n = 8 in each of the four groups; *p* = 0.0048 for Loxp-CRS vs. Loxp-No-CRS, *p* = 0.0345 for Loxp-CRS vs. CKO-CRS). **O** OFT in HDAC9 CKO mice subjected to CRS (n = 8 in each of the four groups). *p < 0.05, **p < 0.01, ***p < 0.001. Differences were assessed for significance using a two-tailed *t* test in panels **B**–**E**, **G**–**J** and **L**; or one-way ANOVA with Tukey’s post hoc test in panels **M**–**O** after adjusting for multiple comparisons. Error bars represent the standard error of the mean

Injection of AAV-HDAC9, but not AAV-CON, into the hippocampus of CaMKIIα-Cre mice (Fig. 2A) led to HDAC9 overexpression in hippocampal neurons after four weeks (Fig. 2B). AAV-HDAC9 prolonged immobility time in the FST and TST (Fig. 2C) and reduced sucrose preference (Fig. 2D), without altering locomotor ability (Fig. 2E).

Together, these results suggest that the HDAC9 level in the hippocampus is elevated in mice with depressive-like behavior and that this increase in HDAC9 expression is sufficient to induce depressive behaviors, indicating that HDAC9 can contribute to depressive disorders.

### HDAC9 deficiency in hippocampal neurons reverses depressive behaviours in CRS mice

To knockdown HDAC9 in hippocampal neurons, we injected AAV9-CaMKIIα-eGFP-3xFLAG-miR30shRNA (HDAC9) (AAV-shHDAC9) or AAV9-CaMKIIα-eGFP (AAV- shCON) into the hippocampus of WT mice and then exposed the mice to CRS (Fig. 2F). The protein expression of HDAC9 was significantly decreased after administration of AAV-shHDAC9 (Fig. 2G). Immobility time in the FST and TST was decreased in mice injected with AAV-shHDAC9 and exposed to CRS compared to mice injected with AAV-shCON and exposed to CRS (Fig. 2H). Furthermore, mice exposed to CRS and injected with AAV-shHDAC9 showed a significantly higher preference for sucrose solution (Fig. 2I), and there was no significant difference in locomotor ability between these two groups of mice (Fig. 2J). Together, these results suggest that HDAC9 deficiency in the hippocampal region can relieve CRS-induced depressive behaviours.

To further investigate the functions of HDAC9, we generated HDAC9 CKO (HDAC9^flox/flox^/CaMKIIα cre) mice, in which HDAC9 was knocked out in forebrain neurons (Fig. 2K). Western blot analysis showed that HDAC9 levels in the hippocampus were significantly lower in HDAC9 CKO mice than in HDAC9 Loxp mice (Fig. 2L). Furthermore, immunofluorescence showed that HDAC9 expression was significantly reduced in hippocampal Camk2a+ neurons in HDAC9 CKO mice (Fig. S4A). There were no differences in body weight, organ weight, or hippocampus weight between 8-week-old HDAC9 CKO and control mice (Fig. S4B–D). Next, a series of behavioural tests was performed to investigate the impact of HDAC9 deficiency on the effect of CRS. After exposure to CRS, HDAC9 CKO mice (CKO-CRS) showed a shorter immobility time and a higher preference for sucrose solution than control mice (Loxp-CRS) (Fig. 2M, N), although there was no apparent difference in locomotor ability among four groups (Fig. 2O). Together, these results indicate that HDAC9 knockout in hippocampal neurons selectively mitigates depressive behaviours in mice exposed to CRS without affecting motor ability.

### HDAC9 reduces dendritic spine density of hippocampal neurons

**Fig. 3:**
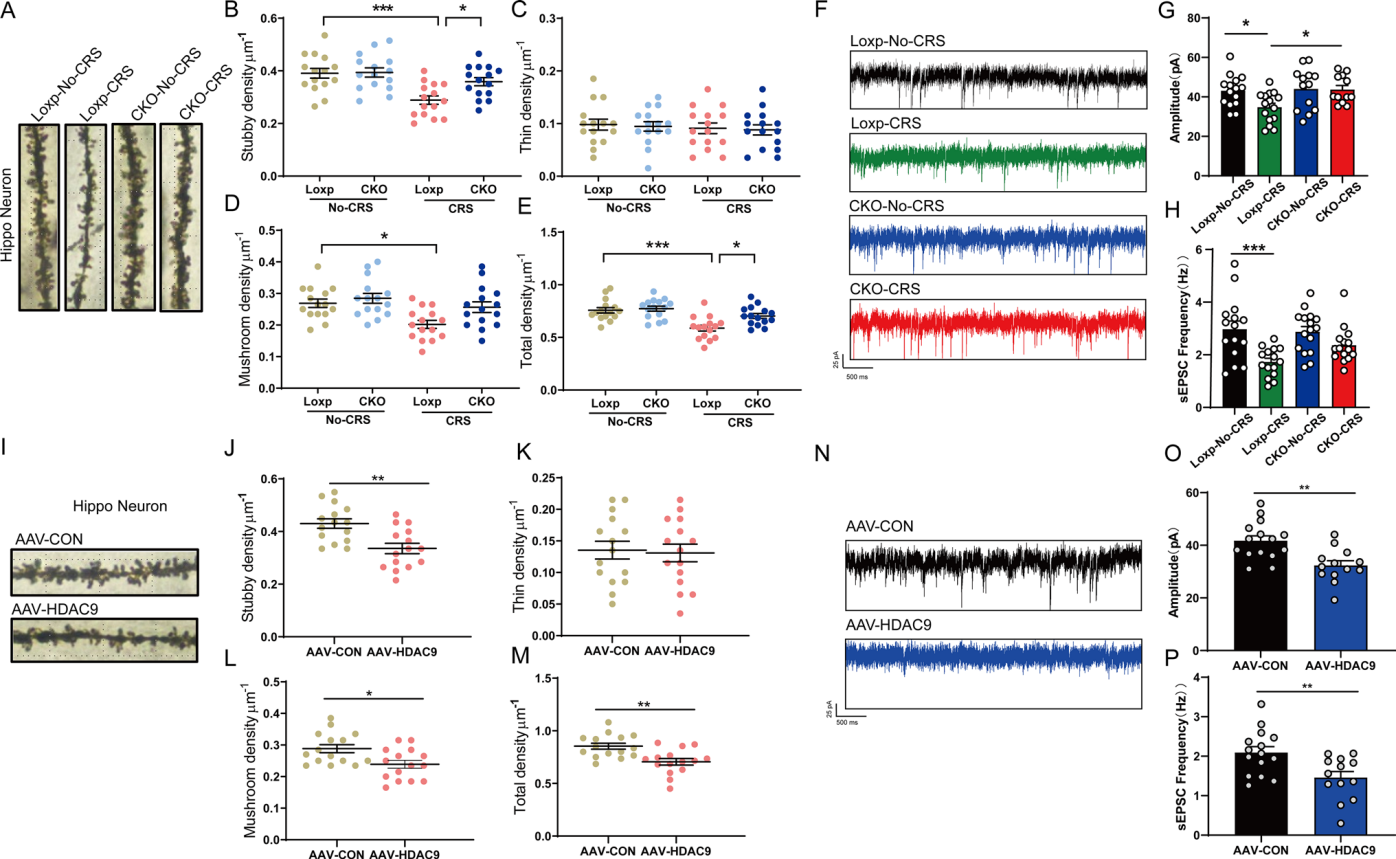
HDAC9 in the hippocampus decreases dendritic spine density and excitability of pyramidal neurons. **A** Representative photomicrographs of dendritic spines from CA1 neurons in HDAC9 Loxp mice (Loxp-No-CRS), HDAC9 Loxp mice exposed to CRS (Loxp-CRS), HDAC9 CKO mice (CKO-No-CRS), and HDAC9 CKO mice exposed to CRS (CKO-CRS). **B** Stubby spine density in dendrites of CA1 neurons (n = 15 spines from each group, *p* = 0.0005 for Loxp-CRS vs. Loxp-No-CRS, *p* = 0.0255 for Loxp-CRS vs. CKO-CRS). **C** Thin spine density in dendrites of CA1 neurons (n = 15 spines from each group). **D** Mushroom spine density in dendrites of CA1 neurons (n = 15 spines from each group, *p* = 0.0111 for Loxp-CRS vs. Loxp-No-CRS). **E** Total spine density in dendrites of CA1 neurons (n = 15 spines from each group, *p* < 0.0001 for Loxp-CRS vs. Loxp-No-CRS, *p* = 0.0111 for Loxp-CRS vs. CKO-CRS). **F** Whole-cell voltage-clamp recordings of sEPSCs in CA1 neurons. **G** Quantification of the average amplitude of sEPSCs in neurons from the four groups (n = 12–15 neurons; three mice per group; *p* = 0.044 for Loxp-CRS vs. Loxp-No-CRS, *p* = 0.0414 for Loxp-CRS vs. CKO-CRS). **H** Quantification of the average frequency of sEPSCs in neurons from the four groups (n = 15–16 neurons; three mice per group; *p* = 0.0008 for Loxp-CRS vs. Loxp-No-CRS). **I** Representative photomicrographs of dendritic spines from CA1 neurons in mice infected with AAV-HDAC9 or AAV-eGFP. **J** Stubby spine density in dendrites of CA1 neurons (n = 15 spines from each group, *p* = 0.0014). **K** Thin spine density in dendrites of CA1 neurons (n = 15 spines from each group). **L** Mushroom spine density in dendrites of CA1 neurons (n = 15 spines from each group, *p* = 0.0108). **M** Total spine density in dendrites of CA1 neurons (n = 15 spines from each group, *p* = 0.0014). **N** Whole-cell voltage-clamp recordings of sEPSCs in CA1 neurons in the hippocampus of AAV-HDAC9-injected mice and AAV-eGFP-injected mice. **O** The amplitude of sEPSCs in AAV-HDAC9-injected mice compared to AAV-eGFP-injected mice (n = 13–15 neurons; three mice per group; *p* = 0.0012). **P** The frequency of sEPSCs in AAV-HDAC9-injected mice compared with AAV-eGFP-injected mice (n = 13–15 neurons; three mice per group; *p* = 0.0060). *p < 0.05, **p < 0.01, ***p < 0.001. Two-tailed t test for **J**–**M** and **O**–**P**; one-way ANOVA with Tukey’s post hoc test for **B**–**E** and **G**–**H**. Adjustments were made for multiple comparisons tests. Error bars represent the standard error of the mean

We performed Golgi staining to analyse the spine density of neurons in the hippocampus after mice were exposed to CRS. The stress regime reduced the densities of stubby and mushroom spines, but not of thin spines (Fig. 3A–E). These changes were not observed in HDAC9 CKO mice after CRS (Fig. 3A–E). The effects of CRS on densities of stubby and mushroom dendritic spines were replicated by overexpressing HDAC9 in CaMKIIα-Cre mice in the absence of CRS (Fig. 3I–M).

### HDAC9 reduces the excitability of hippocampal neurons

To further examine whether HDAC9 inhibits synaptic transmission, we recorded spontaneous excitatory postsynaptic currents (sEPSCs) in hippocampal neurons (Fig. 3F). We found that the amplitude and frequency of sEPSCs were significantly decreased in mice exposed to CRS compared to mice that experienced no CRS (Fig. 3G, H). Interestingly, the amplitude of sEPSCs in HDAC9 CKO mice exposed to CRS were higher than those in HDAC9 Loxp mice exposed to CRS (Fig. 3G, H).

In addition, we examined the effect of AAV-HDAC9 on sEPSCs in hippocampal neurons. Similar to CRS, HDAC9 overexpression significantly decreased the frequency and amplitude of sEPSCs in CaMKIIα-Cre mice (Fig. 3N–P).

### HDAC9 binds to, and deacetylates, ANXA2 to inhibit its ubiquitination-mediated degradation

We performed immunoprecipitation-mass spectrometry to identify downstream proteins through which HDAC9 may exert its pro-depressive effects. We compared proteins that co-immunoprecipitated with HDAC9 in the hippocampus of mice exposed or not to CRS. We identified 29 proteins whose levels differed between the two conditions, and we identified three (Myh10, Myh9, and ANXA2) whose levels in immunoprecipitates were at least 1.5-fold higher from CRS animals than control ones (Table S2). Among these three proteins, only ANXA2 is considered to be closely related to depressive episodes[20, 21] and thus may serve as a downstream target of HDAC9 in regulating depressive behaviour. We performed Gene Ontology enrichment analysis to predict biological process associated with HDAC9-binding proteins (Fig. S5).

**Fig. 4:**
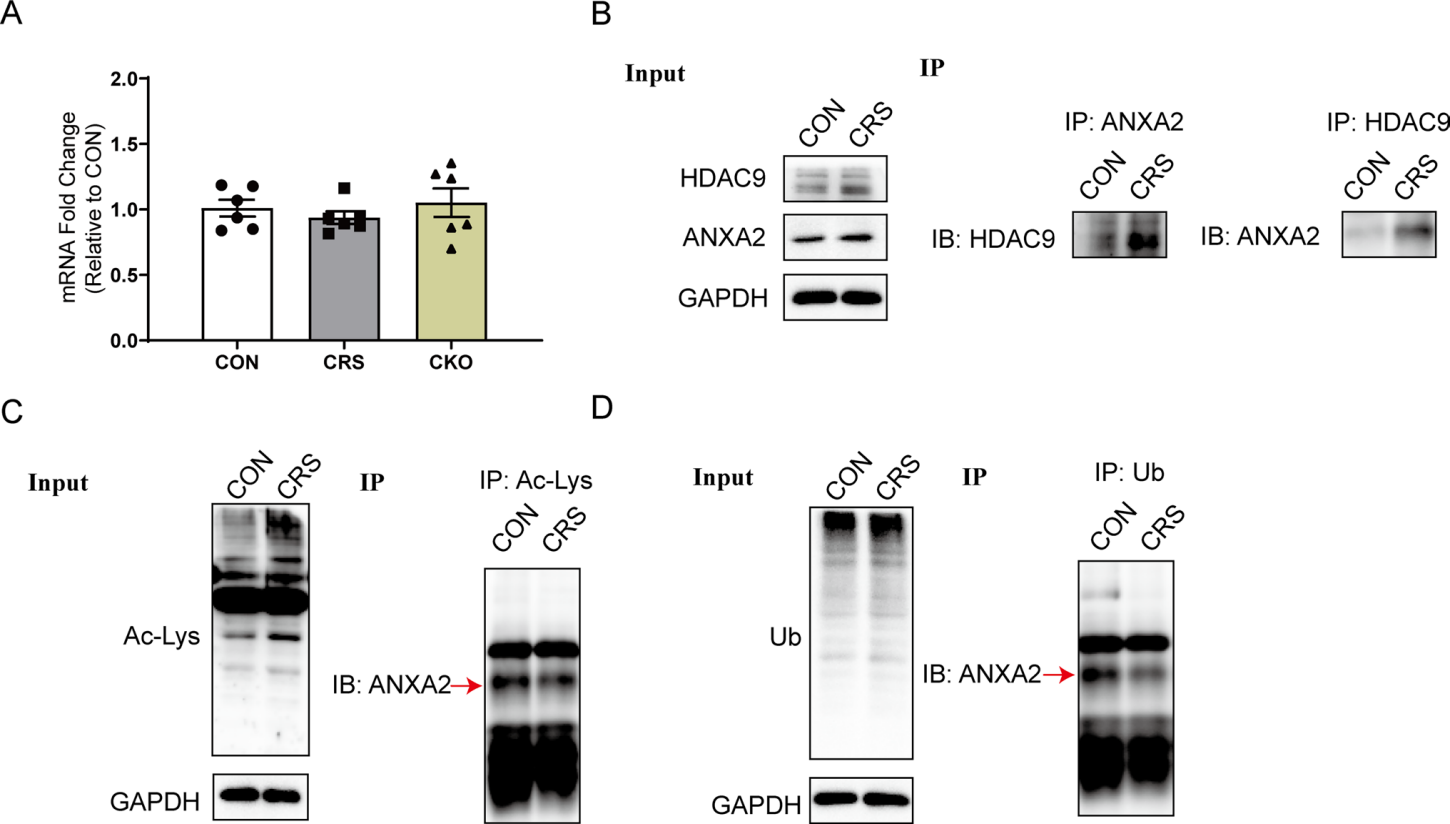
HDAC9 deacetylates ANXA2 to inhibit its ubiquitination-mediated degradation. **A** Real-time quantitative PCR analysis of ANXA2 in mice exposed to chronic restraint stress (CRS) or not (CON), and in HDAC9 conditional knockout (CKO) mice (n = 6 for each group). **B** Co-immunoprecipitation analysis showed that ANXA2 specifically binding to HDAC9 in hippocampus of CRS mice. **C** Immunoprecipitation analysis showed that decreased levels of ANXA2 were pulled down with an antibody against Acetyl-Lys in hippocampus of CRS mice. **D** Immunoprecipitation analysis showed that decreased levels of ANXA2 were pulled down with an antibody against ubiquitin in hippocampus of CRS mice

We then aimed to confirm ANXA2 as the downstream target of HDAC9. RT–PCR showed that ANXA2 mRNA levels were similar in WT mice, WT mice exposed to CRS, and HDAC9 CKO mice (Fig. 4A), suggesting that ANXA2 mainly underwent posttranslational modification. After we observed direct binding between HDAC9 and ANXA2 in hippocampus of CRS mice by co-immunoprecipitation assay (Fig. 4B), we wondered whether HDAC9 might deacetylate ANXA2. Indeed, fewer ANXA2 was pulled down by an antibody against acetylated-Lys in hippocampus of CRS mice comparing to control mice, suggesting that HDAC9 can directly deacetylate ANXA2 (Fig. 4C).

How does the deacetylation of ANXA2 affect its protein content? The ubiquitin–proteasome pathway (UPP) is the main pathway through which ubiquitinated proteins are processed. This pathway is responsible for the degradation of 80–85% of proteins in eukaryotic organisms[22], and it has been reported that ANXA2 can also be degraded by ubiquitination modification to reduce its content [23]. By co-immunoprecipitation assay, we found that the amount of ANXA2 proteins pulled down by an antibody against ubiquitin was significantly reduced in hippocampus of CRS mice comparing to control mice, illustrating that HDAC9 can inhibit the ubiquitination-mediated degradation of ANXA2 (Fig. 4D).

### ANXA2 contributes to depressive-like behaviours in CRS mice

**Fig. 5:**
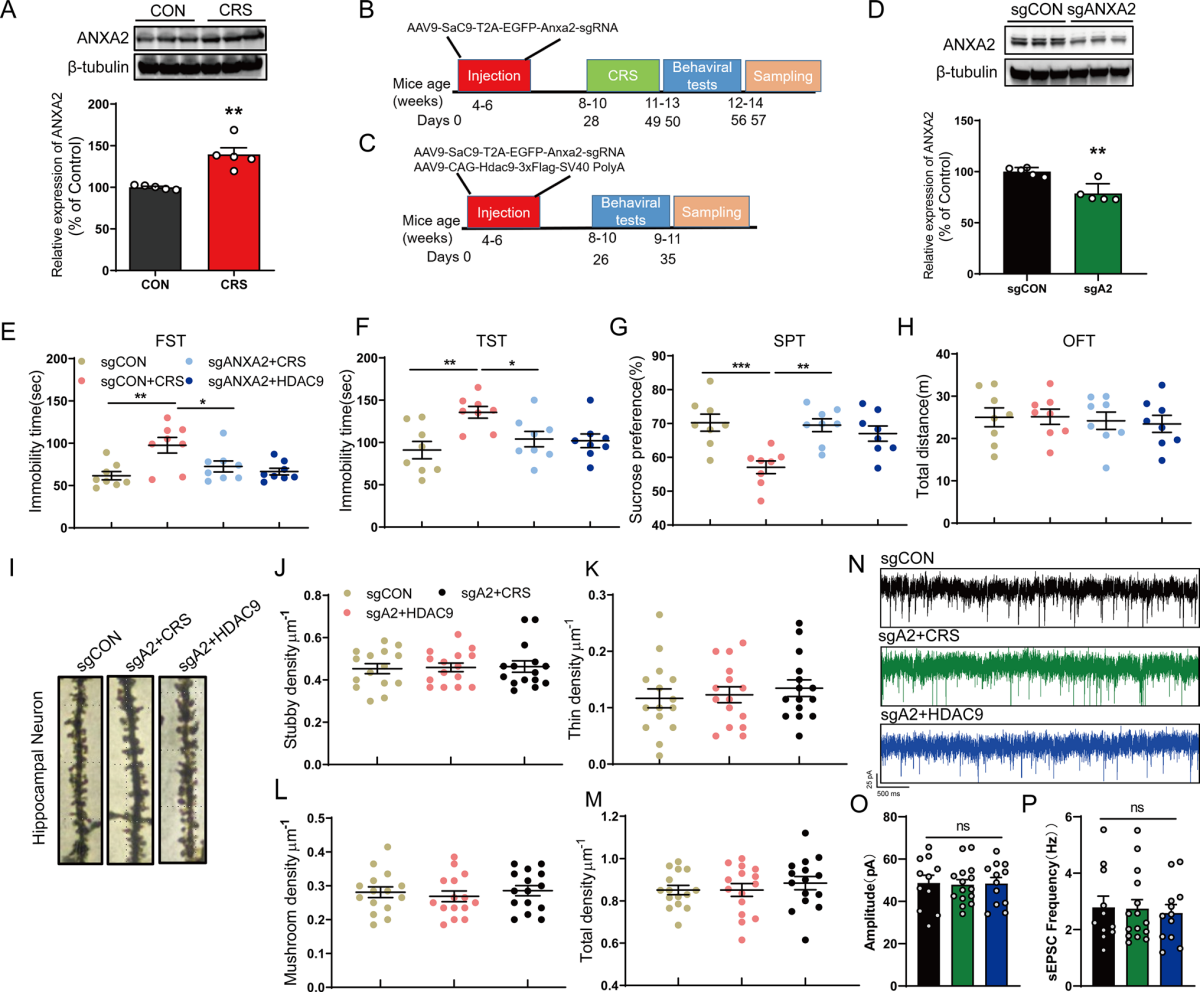
Downregulation of ANXA2 rescues depression-like behaviours and spine loss induced by chronic restraint stress (CRS) or HDAC9. **A** Western blot analysis of ANXA2 expression in the hippocampus of CRS mice and control (CON) mice (n = 5 mice per group; *p* = 0.0013). **B**, **C** Timeline of adeno-associated virus (AAV) injection, behavioural tests, and western blotting. **D** Western blotting of ANXA2 in the hippocampus of mice that received stereotaxic administration of AAV-sgCON or AAV-sgANXA2 (n = 5 mice per group; *p* = 0.0018). **E** Immobility time in the FST. *p* = 0.0027 for sgCON vs. sgCON + CRS, *p* = 0.0106 for sgCON + CRS vs. sgANXA2 + CRS. **F** Immobility time in the TST; *p* = 0.0058 for sgCON vs. sgCON + CRS, *p* = 0.0494 for sgCON + CRS vs. SgANXA2 + CRS). **G** Sucrose consumption in the sucrose preference test (SPT; *p* = 0.0009 for sgCON vs. sgCON + CRS, *p* = 0.0018 for sgCON + CRS vs. SgANXA2 + CRS). **H** Total distance travelled in the OFT. For all the behavioural tests (**E**–**H**), n = 8 in each of the four groups. **I** Representative images of dendritic segments of hippocampal CA1 neurons in mice injected with AAV-eGFP (sgCON), mice exposed to CRS and injected with AAV-sgANXA2 (sgA2 + CRS), and mice injected with AAV-sgANXA2 and AAV-HDAC9 (sgA2 + HDAC9). **J** Stubby spine density in dendrites of CA1 neurons. **K** Thin spine density in dendrites of CA1 neurons. **L** Mushroom spine density in dendrites of CA1 neurons. **M** Total spine density in dendrites of CA1 neurons. In panels **J**–**M**, n = 15 spines from each group. **N** Whole-cell voltage-clamp recordings of sEPSCs in CA1 neurons of mice injected with AAV-eGFP (sgCON), mice exposed to CRS and injected with AAV-sgANXA2 (sgA2 + CRS), and mice injected with AAV-sgANXA2 and AAV-HDAC9 (sgA2 + HDAC9). **O** The amplitude of sEPSCs in hippocampal neurons from the three groups (n = 11–15 neurons; three mice per group). **P** The frequency of sEPSCs in hippocampal neurons from the three groups (n = 11–15 neurons; three mice per group). *p < 0.05, **p < 0.01, ***p < 0.001. Two-tailed t test for **A** and **D**; one-way ANOVA with Tukey’s post hoc test for others. Adjustments were made for multiple comparisons tests. Error bars represent the standard error of the mean

Here, we found that ANXA2 expression in the hippocampus was higher in mice exposed to CRS than in control mice (Fig. 5A). To determine whether ANXA2 contributes to CRS-induced depressive behaviours, we injected AAV-sgANXA2 or AAV-sgCON into the hippocampus of CaMKIIα Cre mice (Fig. 5B). Furthermore, to further clarify whether HDAC9 triggers depressive behaviours by regulating ANXA2, we injected AAV-sgANXA2 and AAV-HDAC9 into the hippocampus of CaMKIIα-Cre mice (Fig. 5C). We found that ANXA2 expression was decreased in the hippocampus of mice injected with AAV-sgANXA2 compared to those of control mice (Fig. 5D). Regarding depressive behaviours, immobility time in the FST and TST was decreased in mice exposed to CRS and injected with AAV-sgANXA2 compared to mice exposed to CRS and injected with AAV-sgCON (Fig. 5E, F). Mice injected with AAV-sgANXA2 and AAV-HDAC9 and mice injected with AAV-sgCON showed a similar immobility time (Fig. 5E, F). Mice exposed to CRS and injected with AAV-sgANXA2 showed a significantly higher preference for sucrose solution than mice exposed to CRS and injected with AAV-sgCON (Fig. 5G). A similar preference for sucrose solution was observed in mice injected with AAV-sgANXA2 and AAV-HDAC9 and mice injected with AAV-sgCON (Fig. 5G). Indeed, there was no significant difference in locomotor ability among these four groups of mice (Fig. 5H). In summary, these results indicate that ANXA2 deficiency in the hippocampus can alleviate CRS- or HDAC9-induced depressive behaviours, suggesting that ANXA2 may contribute to CRS-induced depressive behaviours.

### ANXA2 mediates the effects of CRS and HDAC9 on dendritic spine density and synaptic function

To clarify how ANXA2 alleviates depression-like behaviours caused by CRS or HDAC9 overexpression, we examined whether ANXA2 plays a role in dendritic spine development in the hippocampus. Using Golgi staining, we found that the dendritic spine density in mice exposed to CRS and HDAC9-overexpressing mice was similar to that in control mice after treatment with ANXA2 sgRNA (Fig. 5I–M). To assess synaptic function in these mice, we recorded sEPSCs in hippocampal neurons from the three groups of mice (WT mice injected with sgCON, mice exposed to CRS and injected with ANXA2 sgRNA, and HDAC9 OE mice injected with ANXA2 sgRNA) (Fig. 5N). We found that the amplitude and frequency of sEPSCs in these mice were not significantly different (Fig. 5O, P), suggesting that knockdown of ANXA2 prevents impairment of synaptic function induced by CRS and HDAC9.

## Discussion

Here we provide evidence in vivo that HDAC9 contributes to depressive-like behaviours. CRS upregulates the enzyme specifically within hippocampal neurons in mice, which in turn reduces the density of particular spine subpopulations as well as the excitability of hippocampal neurons. Knockdown or knockout of HDAC9 in hippocampal regions mitigated the depressive-like behaviours induced by CRS, as did knockdown of the downstream target of HDAC9, ANXA2. These findings identify HDAC9 and ANXA2 as potental therapeutic targets against MDD.

The etiology of depression is complex, although genetic factors may play a significant role, non-genetic factors such as stress also play an important role in the occurrence of depression [24]. These non-genetic factors may induce changes in gene expression in the brain through epigenetics mechanisms, such as histone modification, DNA methylation, etc., thus leading to changes in neuronal plasticity in areas related to disease pathogenesis [25, 26]. Studies believe that histone acetylation is an important post-transcriptional modification, and the structural remodeling of chromatin caused by histone acetylation is an important mechanism of gene transcriptional regulation, which is mainly regulated by two enzymes with opposite roles, namely histone acetyltransferases (HATs) and histone deacetylases (HDACs) [27]. In normal physiology, they are in a dynamic equilibrium process, regulating histone acetylation modification, ensuring the orderly expression of genes, so as to ensure the balance of various functions of the body [28]. In recent years, studies have found that histone acetylation may be involved in a variety of mental diseases [29]. For example, after the mice were given sodium butyrate, a broad-spectrum inhibitor of HDACs, their immobility time was significantly reduced in the tail suspension experiment, suggesting that histone deacetylation plays a role in the occurrence of depression [12].

Our study found that after experiencing CRS, HDAC9 specifically increased in the hippocampus of mice, while other HDACs did not show significant changes, suggesting that HDAC9 is involved in CRS induced depressive behavior. However, previous studies have found that other HDACs are also involved in depressive episodes or related to anti-depressant drugs, such as: 1. Antidepressant effects of fluoxetine in an LPS-induced mouse model of depression may involve HDAC1-eEF2 related neuroinflammation and synaptogenesis [30]; 2. TRPV1 Regulates Stress Responses induced by chronic unpredictable stress through HDAC2 [31]; 3. Inhibition of HDAC3 using RGFP966 could serve as a potential treatment strategy for depression [32]; 4. Hippocampal HDAC4 contributes to postnatal fluoxetine-evoked depression-like behavior [33]; 5. Chronic social defeat stress and antidepressant induced changes in HDAC5 and Sirt2 affect synaptic plasticity [34]; 6. Alleviation of depression-like behavior in a cystic fibrosis mouse model by HDAC6 depletion [35]; 7. Depressive-like behaviors induced by chronic social defeat stress are associated with HDAC7 reduction in the nucleus accumbens [36]. Of course, the depression models used in these previous studies are different, and the targeted brain regions and cell types are also different. This may explain why we believe that hippocampal neuron HDAC9 plays a key role in CRS induced depression rather than other HDACs.

Interestingly, during the experiment, we also found some HDAC9 upregulation in glial cells in the mouse hippocampus after experiencing chronic restraint stress, but this is not universal, most microglia and astrocytes do not have HDAC9 expression, which may be related to the heterogeneity of cells. In the future, we will focus on these glial cells cells for research and explore their role in chronic restraint stress. Due to the low proportion of these glial cells, we believe that under our experimental conditions, it is mainly HDAC9 on neurons that is regulated by chronic restraint stress.

Our results are consistent with previous observations that chronic stress induces depressive episodes which is associated with dendritic spine damage and decreased activity of hippocampal neurons [37–40]. HDACs are involved in dendritic spine development and may regulate synaptic plasticity [41, 42], and the present work specifically identifies HDAC9 as a regulator of dendritic spine density and excitatory synaptic transmission in hippocampal neurons. These findings are consistent with the proposal that HDAC9 helps regulate dendritic growth and memory formation [43].

HDAC9 and other class II HDACs can inhibit gene transcription by binding to various transcription factors [44, 45], and several non-histone substrates of class II HDACs have been described [4]. Little is known about substrates of HDAC9, and here we identify ANXA2 as one. ANXA2 is known to be expressed in the hippocampus [46], and to be essential for the survival of developing cortical neurons and neurite outgrowth [47]. Although there is currently a lack of clear research on ANXA2 in depression, p11 is considered a key targets in depression research. Meanwhile, the components of the p11/ANXA2 protein complex stabilize each other in cells [20], and the protein levels of p11 decreased in various tissues from ANXA2 KO mice [48], suggest that ANXA2 may play a role in depression through p11. However, some studies from the Yong Kim’s group found that reduced levels of ANXA2 increase depression [20, 49, 50]. This contradicts our research findings that elevated ANXA2 expression is associated with depression. The possible explanations were that the animal model of depression used are different, as are the regions and cell types examined.

Previous studies have shown that ANXA2 is activated mainly by phosphorylation and degraded by ubiquitination, but there are few reports about other regulatory mechanisms [51, 52]. Here, co-immunoprecipitation combined with western blotting showed that the acetylation level of ANXA2 decreased in hippocampus of CRS mice, suggesting that HDAC9 directly deacetylates ANXA2 after CRS. Regulation of protein acetylation can affect ubiquitination degradation [53], and we demonstrated this for ANXA2: after CRS, HDAC9 deacetylated ANXA2, decreasing the latter’s ubiquitination and inhibiting its degradation. The resulting accumulation of ANXA2 appears to mediate the effects of CRS or HDAC9 overexpression on depressive-like behaviours.

In summary, our findings suggest that HDAC9 in hippocampal neurons may play a role in the pathophysiology of depression and that HDAC9 regulates the acetylation and ubiquitination of ANXA2. However, our study presents some limitations that need to be considered. First, although we verified that HDAC9 is involved in CRS-induced depressive episodes, we cannot exclude that other HDACs also contribute. This should be explored in future work. Second, we used mainly CRS to model depression model, which may not fully represent the human disease. Our findings should be validated in models of unpredictable chronic mild stress and social defeat stress.

Despite these limitations, our research identifies HDAC9 and ANXA2 as potential targets for treating MDD. It should also inspire studies to explore the potential contributions of other class II HDACs in this disorder.

### Supplementary Information

Below is the link to the electronic supplementary material.

Supplementary file1 (DOCX 4558 KB)

## Data Availability

The authors declare that all the data supporting the findings of this study are available within this article, its supplementary information files, or are available from the corresponding author, who has all relevant data, upon reasonable request.

## Abbreviations

ANXA2: Annexin A2
CRS: Chronic restraint stress
CKO: Conditional knockout
FST: Forced swimming test
HDAC: Histone deacetylase
IP–MS: Immunoprecipitation–mass spectrometry
MDD: Major depressive disorder
OFT: Open field test
ShRNA: Short hairpin RNA
SPT: Sucrose preference test
SEPSCs: Spontaneous excitatory postsynaptic currents
TST: Tail suspension test
UPP: Ubiquitin–proteasome pathway
