# Supplementary material for: Upregulation of HDAC9 in hippocampal neurons mediates depression-like behaviours by inhibiting ANXA2 degradation
Source: Cell Mol Life Sci. 2023 Sep 10;80(10):289. doi: 10.1007/s00018-023-04945-y (PMC10493204; doi:10.1007/s00018-023-04945-y)
Supplement: Supplementary file 1 — Supplementary file1 (DOCX 4558 KB) [file 18_2023_4945_MOESM1_ESM.docx]

**Supplemental Information:**

**Table S1：PCR Primers**


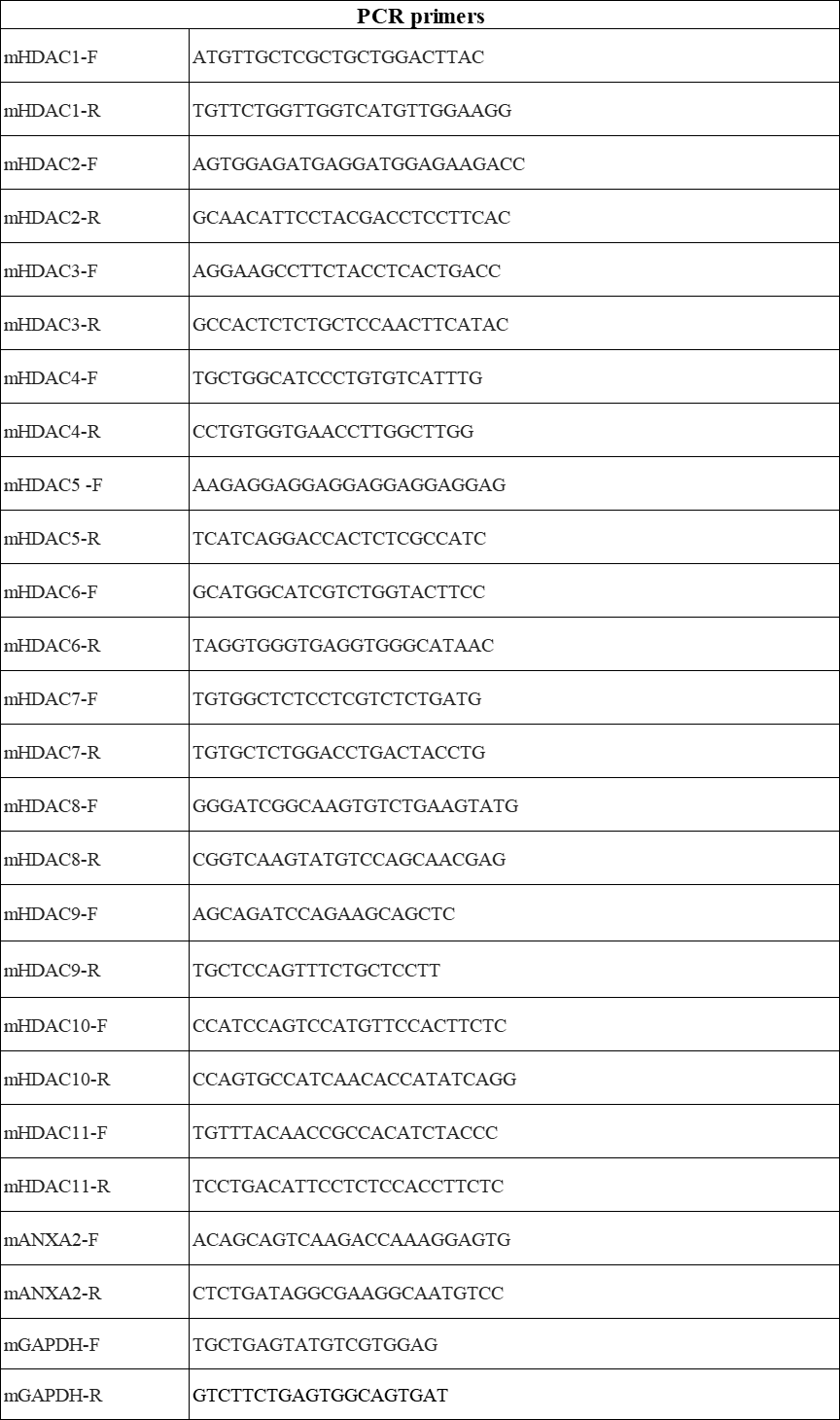


**Table S2：****29 proteins binding to HDAC9 after CRS**

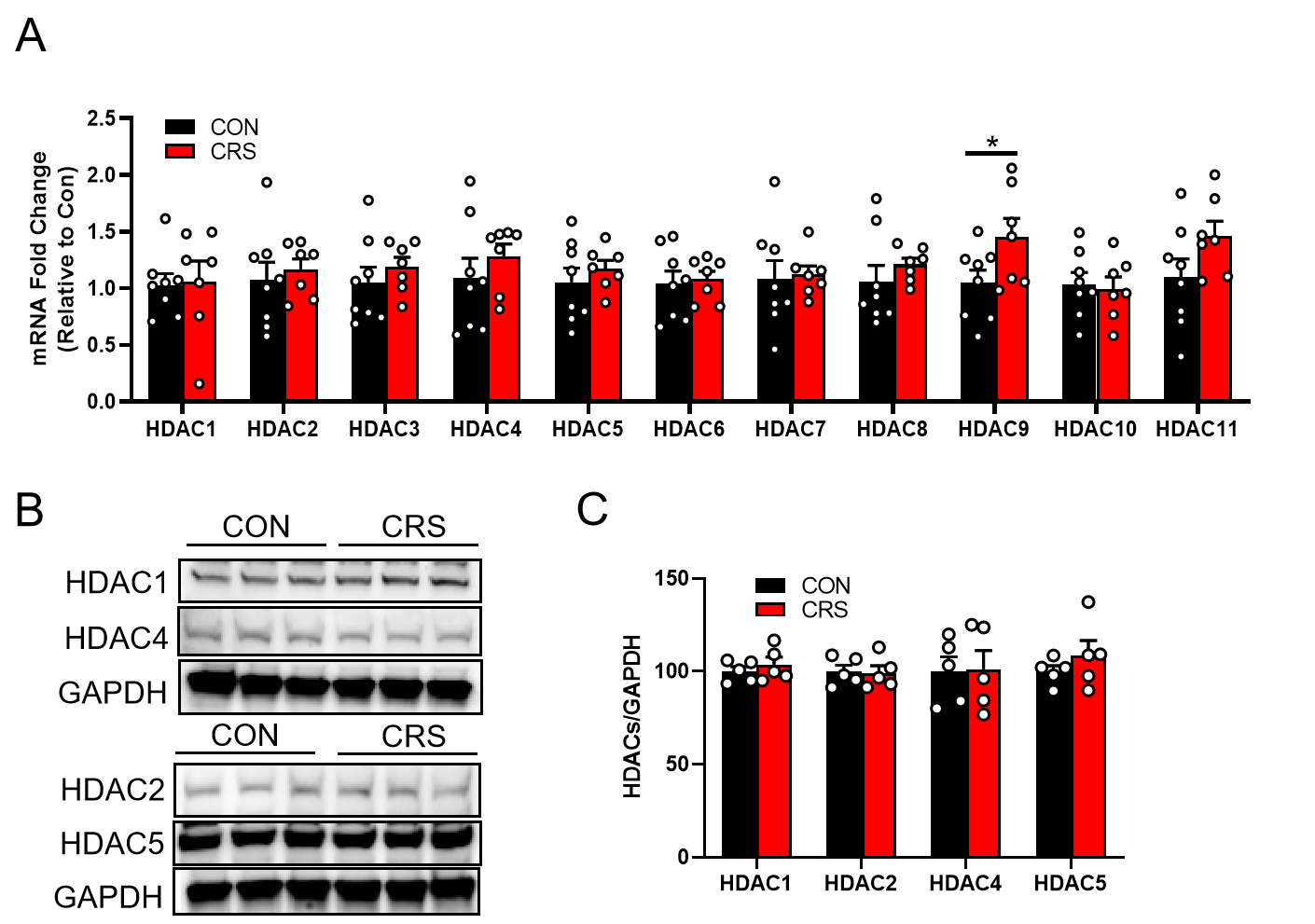


**Figure S1：The contents of HDACs in hippocampus of CRS mice.** (A) Levels of HDAC1, HDAC2, HDAC3, HDAC4, HDAC5, HDAC6, HDAC7, HDAC8, HDAC9, HDAC10 and HDAC11 mRNAs in the hippocampus of WT and CRS mice were assayed by qPCR (n = 8 for CON group，n=7 for CRS group). Data are means±s.e.m. (B) Western blot analysis of HDAC1, 2, 4, and 5 protein levels in the hippocampus of WT and CRS mice. (C) Relative HDACs contents in the hippocampus (n = 5 per group ). Data are mean±s.e.m.


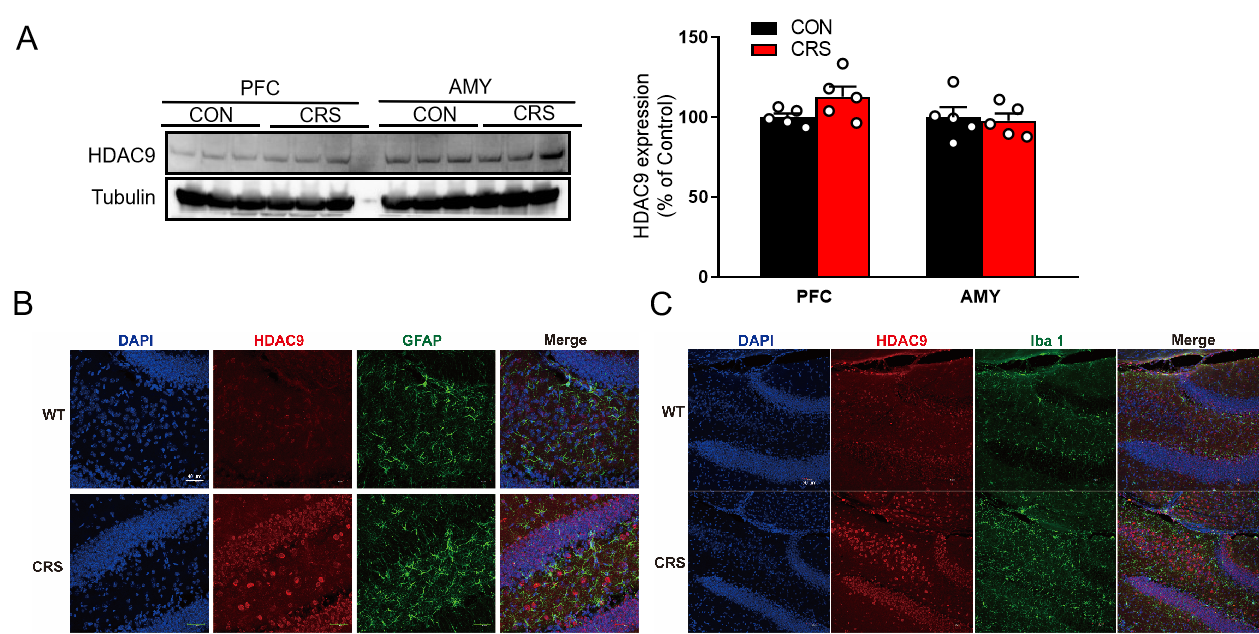


**Figure S2：The content of HDAC9 in depression-related brain regions. (A)** Expression of HDAC9 in prefrontal cortex (PFC) and amygdala (AMY) of CRS mice. n = 5 per group. **(B)** Representative double immunostaining for GFAP and HDAC9 proteins. **(C)** Representative double immunostaining for Iba1 and HDAC9 proteins.Unpaired two-tailed student’s t-test. Adjustments were made for multiple comparisons test. Error bars represent s.e.m.


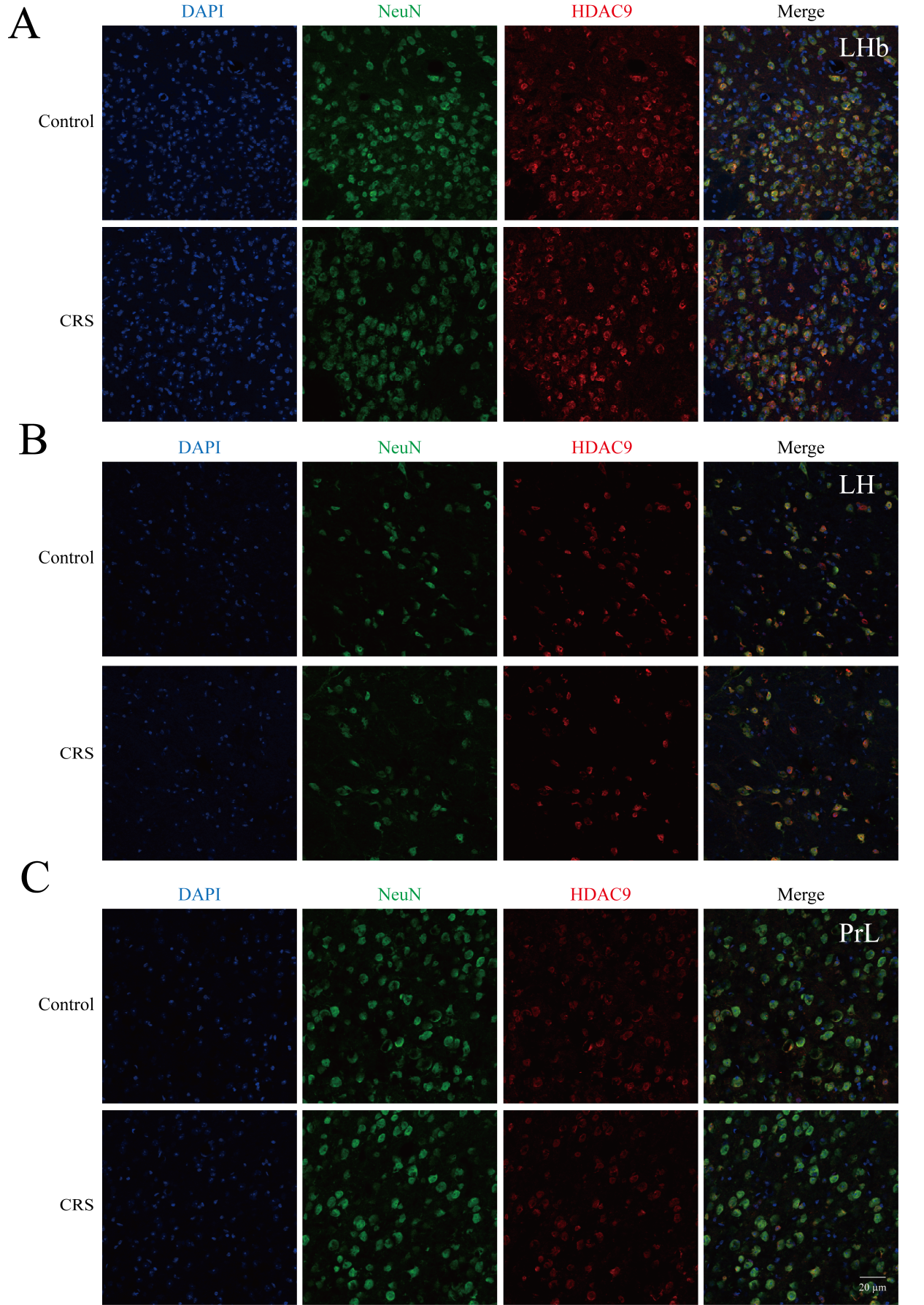


**Figure S3：The distribution of HDAC9 in neurons in depression related brain regions. (A)** Representative double immunostaining for NeuN and HDAC9 proteins in Lateral habenula (LHB). **(B)** Representative double immunostaining for NeuN and HDAC9 proteins in Lateral hypothalamus (LH). **(C)** Representative double immunostaining for NeuN and HDAC9 proteins in Prelimbic cortex (PrL).

**
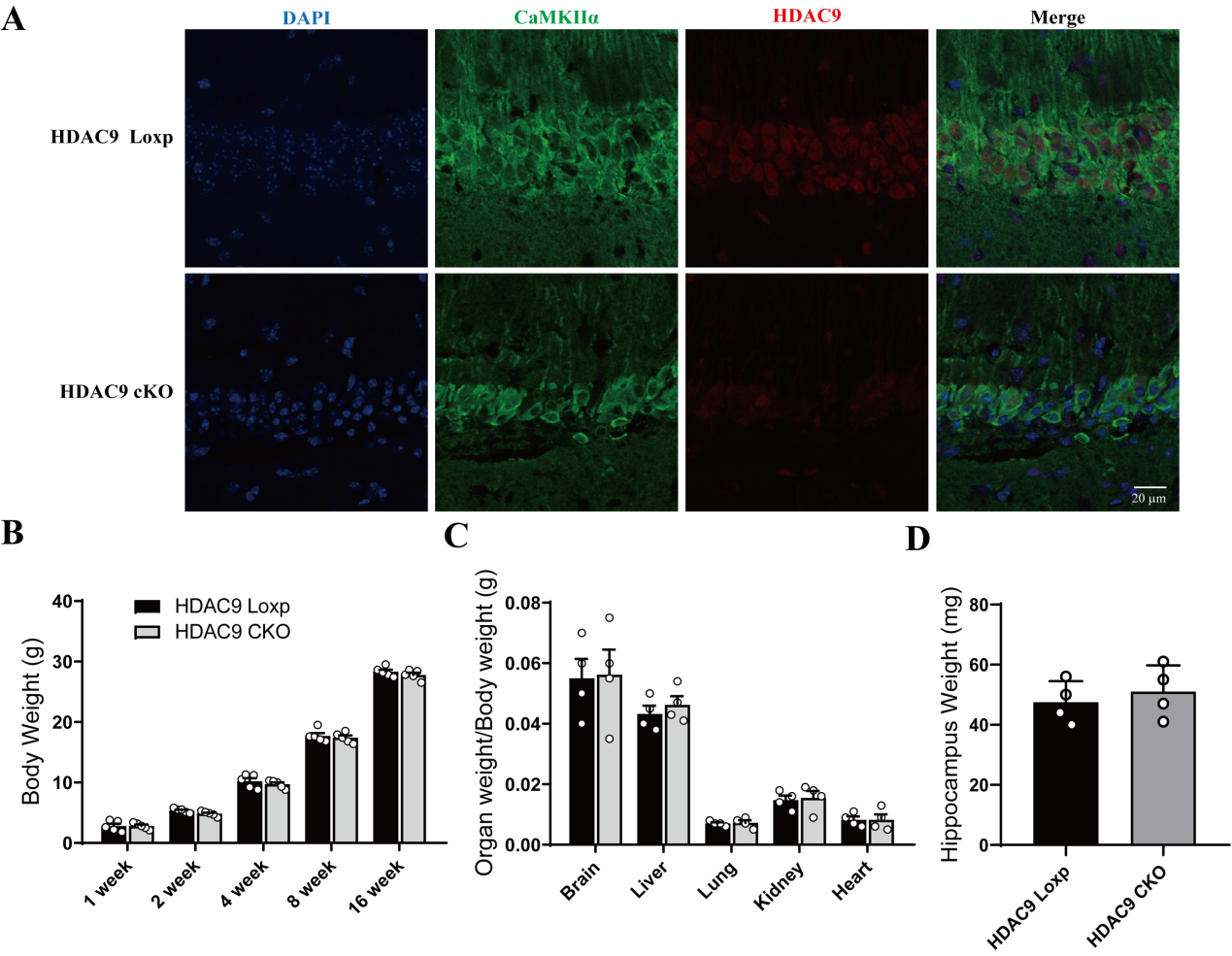
**

**Figure S4：Evaluation of HDAC9 CKO mice. (A)** Representative double immunostaining for HDAC9 and Camk2α in the CA1 of hippocampus from HDAC9 CKO mice or HDAC9 Loxp mice. **(B)** Body weight of HDAC9 CKO mice and HDAC9 Loxp mice were similar during growth (n = 5 mice per group; two-way ANOVA followed by Bonferroni’s post hoc test). **(C)** Organ weight of HDAC9 CKO mice and HDAC9 Loxp mice were similar during growth (n = 4 mice per group; two-way ANOVA followed by Bonferroni’s post hoc test). **(D)** Hippocampus weight of HDAC9 CKO mice and HDAC9 Loxp mice were similar during growth (n = 4 mice per group; unpaired Student’s t-test).


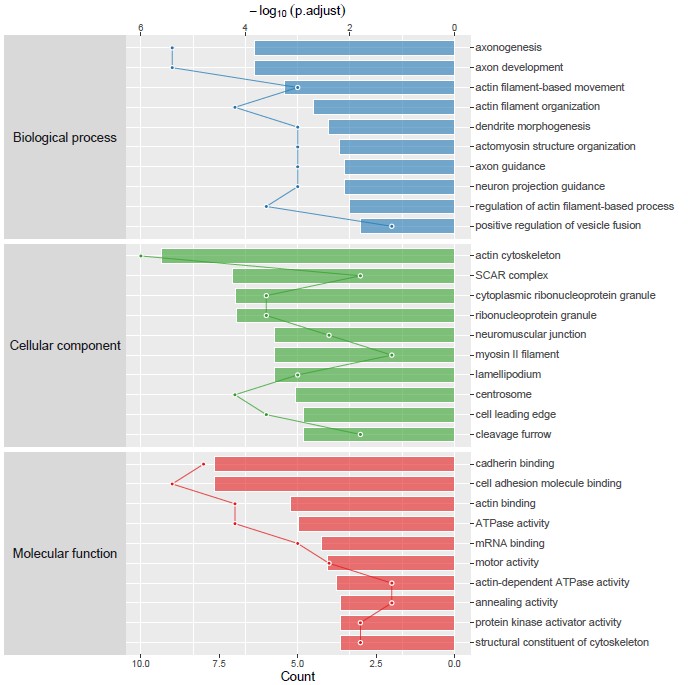


**Figure S5：GO analysis of 29 proteins binding to HDAC9 after CRS**
